# Xylan–Porphyrin Hydrogels as Light-Triggered Gram-Positive Antibacterial Agents

**DOI:** 10.3390/gels9020124

**Published:** 2023-02-02

**Authors:** Abdechakour Elkihel, Charlotte Vernisse, Tan-Sothéa Ouk, Romain Lucas-Roper, Vincent Chaleix, Vincent Sol

**Affiliations:** 1 University Limoges, LABCiS, UR 22722, 87000 Limoges, France; 2 University Limoges, IRCER, UMR CNRS 7315, 87000 Limoges, France

**Keywords:** hydrogel, xylan, photosensitizers, porphyrins, Gram-positive bacteria, photodynamic antimicrobial therapy

## Abstract

In the present work, we report on the synthesis of light-triggered antibacterial hydrogels, based on xylan chains covalently bound to meso-tetra(4-carboxyphenyl)porphyrin (TCPP). Not only does TCPP act as a photosensitizer efficient against Gram-positive bacteria, but it also serves as a cross-linking gelator, enabling the simple and easy building of xylan conjugate hydrogels. The hydrogels were characterized by infrared spectroscopy (ATR-FTIR), scanning electron microscopy (SEM), along with swelling and rheological tests. The antimicrobial activity of the hydrogels was tested under visible light irradiation against two Gram-positive bacterial strains, *Staphylococcus aureus* and *Bacillus cereus*. The preliminary results showed an interesting activity on these bacteria, indicating that these hydrogels could be of great potential in the treatment of skin bacterial infections with this species by photodynamic antimicrobial chemotherapy (PACT).

## 1. Introduction

Photodynamic Antimicrobial ChemoTherapy (PACT) has gained considerable attention in the field of antimicrobial therapy due to its non-invasive nature, broad spectrum of action, and high antimicrobial efficacy against bacteria, viruses, fungi, and parasites, in addition to its low likelihood of resistance development [1,2]. PACT relies on the irradiation of a photosensitizer (PS) with visible light to generate reactive oxygen species (ROS), such as singlet oxygen (^1^O_2_), as cytotoxic agents. The light-generated ROS can react with most biomolecules, such as DNA, proteins, and lipids, and therefore can bring damage to any cellular component, regardless of its structure or function [3,4]. This is mainly why no microbial resistance to PACT has been reported so far. Furthermore, the few microseconds lifetime and the high reactivity of singlet oxygen contribute to shorten its path length and to limit the photooxidative damage caused to the nearby healthy tissues [1,5,6,7]. Among the various dyes that can be used as photosensitizers, porphyrins and other tetrapyrrole molecules, such as phthalocyanines and chlorins, have been widely used in PACT since they possess many desirable properties such as a strong absorption in the visible region and they are known to produce under light a relatively high triplet and singlet state quantum [8,9]. However, many porphyrins and their derivatives are highly hydrophobic and thus are sparingly soluble in biological fluids. Their tendency to aggregate in aqueous media hampers their biodistribution, which results in a loss of photoactivity [10,11]. One way to overcome most of these problems is the incorporation of porphyrins into hydrogels. Indeed, hydrogels are hydrophilic three-dimensional networks formed from natural or synthetic polymers capable of absorbing large amounts of water or biological fluids [12]. Several hydrogels have been recently reported in which PSs were incorporated either by encapsulation or co-polymerization [13,14,15]. The random distribution of PSs in these three-dimensional hydrogel networks actually prevents PS stacking. In addition, the macroporous networks of hydrogels allows molecules and bacteria to freely enter and migrate through these materials [16]. Thus, hydrogels associated with PS could be an attractive strategy for the local treatment of a wound infected with multidrug-resistant bacteria. Indeed, hydrogels allow for a local long-term application of PS and protect the wound from an external infection. This local application limit photoactive damage was caused to the nearby healthy tissues. Moreover, as Ps requires light for activation, a transparent hydrogel is an ideal material to be used as patches or bandages [17]. The incorporation of PSs into a hydrogel can be carried out either by simple non-covalent encapsulation or by covalent bonding; the latter method avoids leakage of PS from the hydrogel and thus prevents a potential loss of activity [16,17,18,19,20]. In our previous work, we presented two methods for the synthesis of transparent xylan-based hydrogels containing covalently or non-covalently attached cationic porphyrins using diethylenetriaminepentaacetic (DTPA) dianhydride as a cross-linking agent [21,22].

In the present article, the photosensitizer, meso-tetra(4-carboxyphenyl)porphyrin (TCPP), serves as a cross-linking node between the xylan chains thanks to its four carboxylic acid functions that can establish ester bonds with xylan hydroxyl groups, leading to the formation of a three-dimensional network characteristic of hydrogel-like structures. Photosensitizers bearing carboxylic functions, as described in our previous work [23], are known to be efficient, along with PACT, against Gram-positive bacteria strains. Several xylan-TCPP hydrogels with different PS/xylan ratios have been obtained and were characterized and studied for their swelling behavior. The hydrogel that showed the highest swelling capacity was selected and its morphology and rheological properties were investigated. The photobactericidal effects of the selected xylan-TCPP hydrogel were evaluated against two Gram-positive bacteria: *Staphylococcus aureus* and *Bacillus cereus*.

## 2. Results and Discussion

### 2.1. Xylan-TCPP Hydrogels: Preparation and Characterization

**Figure 1:**
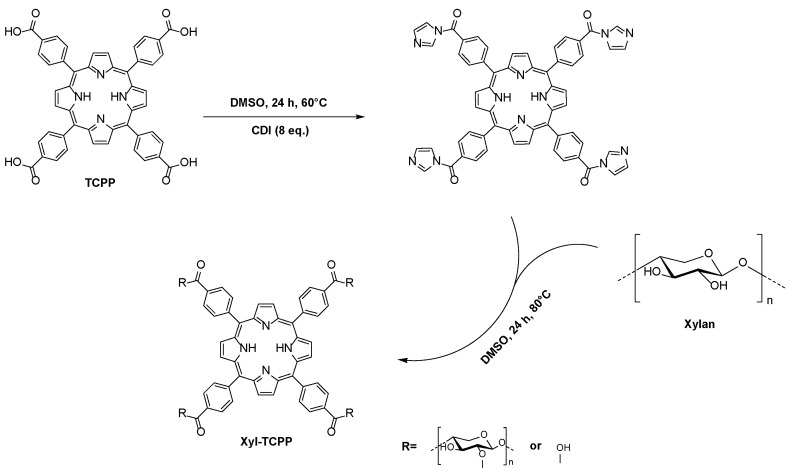
Synthesis of hydrogels by cross-linking xylan with TCPP.

Xylan cross-linking was performed by the esterification of the hydroxyl groups of the xylan backbone using *N,N′*-carbonyldiimidazole (CDI) as a coupling agent. After the activation of the TCPP carboxylic acid functions using 8 eq. of CDI in DMSO for 24 h at 60 °C, xylan, previously dissolved in DMSO, was added and then the reaction was followed for an additional 24 h at 80 °C. The resulting hydrogel was immersed in excess distilled water and allowed to swell for 7 days at room temperature with a daily water change and it was then freeze-dried (Figure 1).

**Figure 2:**
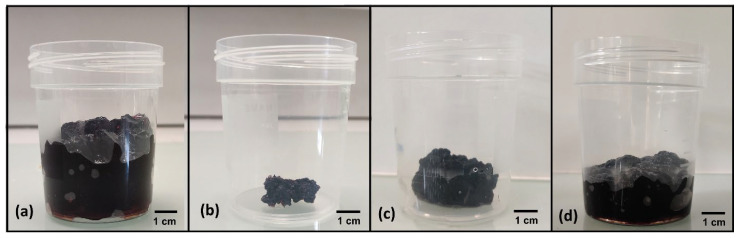
(**a**) xyl-TCPP-1 swollen in water directly after reaction; (**b**) xyl-TCPP-1 after lyophilization; (**c**) xyl-TCPP-1 after 48 h; and (**d**) after 15 days of swelling in water.

Initially, we used 200 mg of TCPP per gram of xylan (i.e., 0.033 eq. per anhydroxylose unit) to obtain the xyl-TCPP-1 hydrogel. After freeze-drying, we noticed that the obtained hydrogel did not fully swell in water. Even after a 15-day long incubation, this hydrogel was unable to absorb as much water as before freeze-drying (Figure 2).

We assumed that hydrogel freeze-drying and the consecutive shrinking promoted the bringing together of the porphyrin residues, enhancing their mutual hydrophobic interactions, and strongly hampering water re-entry into the hydrogel structure. To overcome this problem, we synthesized hydrogels containing lesser amounts of porphyrins. Accordingly, xyl-TCPP-2, and xyl-TCPP-3 were obtained in the presence of, respectively, 0.025 and 0.017 eq. TCPP per anhydroxylose unit.

**Figure 3:**
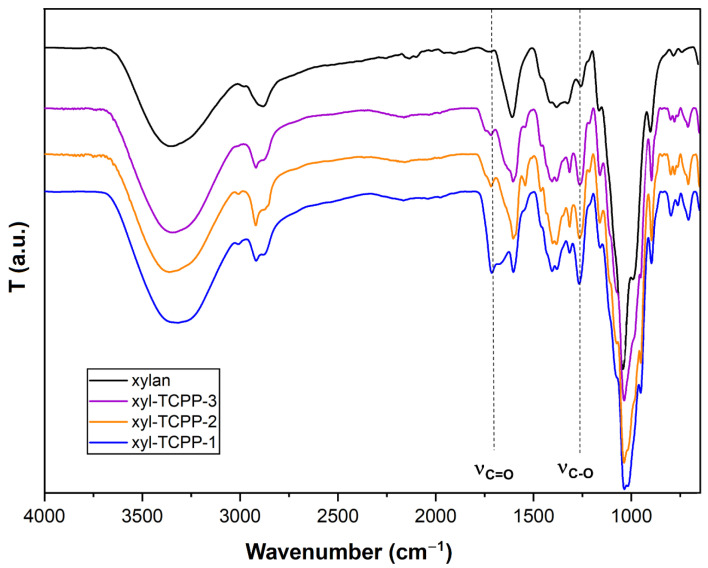
FTIR spectra of native xylan, xyl-TCPP1, xyl-TCPP-2, and xyl-TCPP-3.

The covalent binding between TCPP and xylan was confirmed by FTIR. The corresponding FTIR spectra of native xylan, xyl-TCPP-1, xyl-TCPP-2, and xyl-TCPP-3 are displayed in Figure 3. These compounds and native xylan shared a characteristic absorption band around 3400 cm^−1^, which has been assigned to the stretching vibrations of hydroxyl groups of xylan. There were two characteristic bands, only present on the spectra of xyl-TCPP-1, xyl-TCPP-2, and xyl-TCPP-3, which are assigned to a carbonyl group: a band at 1713 cm^−1^ corresponding to the stretching of the carbonyl group and a band at 1267 cm^−1^ assigned to the stretching of the –C–O– bond. As expected, the higher the amount of TCPP used in hydrogel synthesis, the stronger are these absorption bands.

To determine the amount of TCPP residues in each hydrogel, freeze-dried xyl-TCPP-1, xyl-TCPP-2, and xyl-TCPP-3 hydrogels were immersed in 2 M of aqueous NaOH in order to break up the hydrogel matrix and to hydrolyze the xylan–porphyrin ester bonds. TCPP mass percentages were revealed by measurement of the UV–Vis spectra (410 nm) using a calibration curve developed from different concentrations of TCPP in sodium hydroxyl (2M) solution (Table 1) (Figure S1).

### 2.2. Swelling Properties

**Figure 4:**
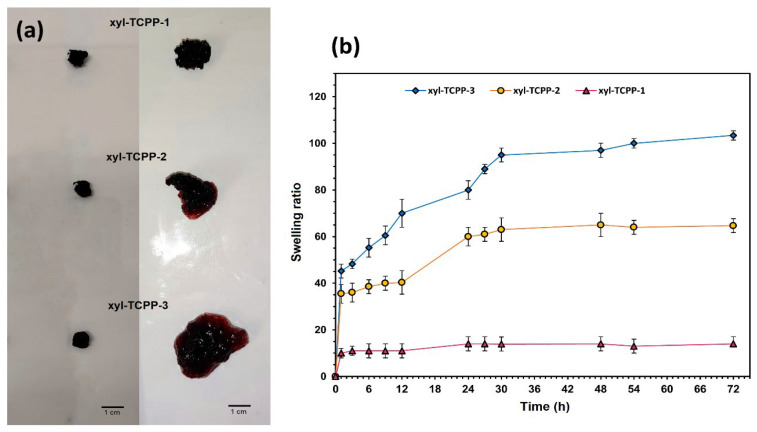
(**a**): freeze-dried xyl-TCPP-1, xyl-TCPP-2, and xyl-TCPP-3, left, before swelling and right, after 72 h immersion in water; (**b**): time course of swelling ratios of xyl-TCPP-1, xyl-TCPP-2, and xyl-TCPP-3 in distilled water.

In order to confirm our hypothesis on the effect of the amount of grafted TCPP on the swelling behavior of the hydrogels, we first followed the swelling time course of the three hydrogels in distilled water at 37 °C. As shown in Figure 4a, hydrogels xyl-TCPP-2 and xyl-TCPP-3 showed a good swelling ability compared with xyl-TCPP-1. The initial swelling ratio of xyl-TCPP-2 and xyl-TCPP-3 is relatively fast (about 35.5 and 45.2, respectively, after one hour), then the swelling ratios continue to increase with time to reach equilibrium after 24 h for xyl-TCPP-2 and after 30 h for xyl-TCPP-3. In contrast, xyl-TCPP-1 showed a low and stable swelling ratio (about 14) throughout the whole experiment (Figure 4b).

**Figure 5:**
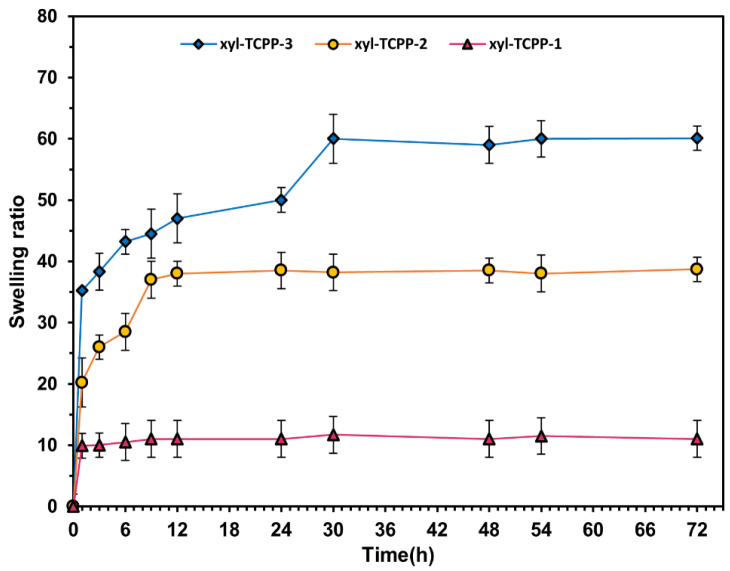
Time course of swelling ratios of xyl-TCPP-1, xyl-TCPP-2, and xyl-TCPP-3 in PBS.

Since our biological evaluations are being performed in PBS (phosphate-buffered saline, pH = 7.4), we tested the swelling ratios of the three hydrogels in PBS (Figure 5). These tests showed again that the lesser grafted the TCPP is, the better is the swelling ability of the hydrogel. Xyl-TCPP-3, which presents the best swelling ability in PBS, was chosen for the rest of this study. Thus, Xyl-TCPP-3 had a swelling ratio about 100% after 30 min. That means it can absorb about one time his own weight of water or wound exudate and could be a good candidate for health’s application.

### 2.3. Rheological Behavior of the Hydrogel

**Figure 6:**
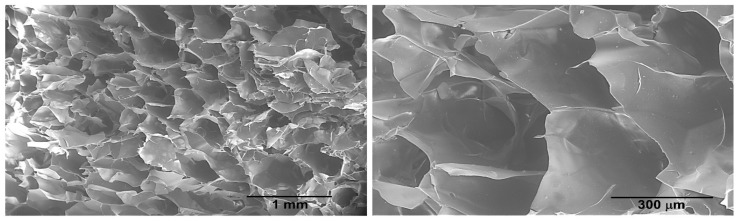
SEM images of cross sections of lyophilized xyl- TCPP-3.

SEM images of freeze-dried xyl-TCPP-3 (Figure 6) show an interconnected porous, honeycomb-like structure with a pore diameter comprised between 200 and 400 µm that may promote ^1^O_2_ transport and also facilitate the entry of bacteria into the hydrogel structure.

**Figure 7:**
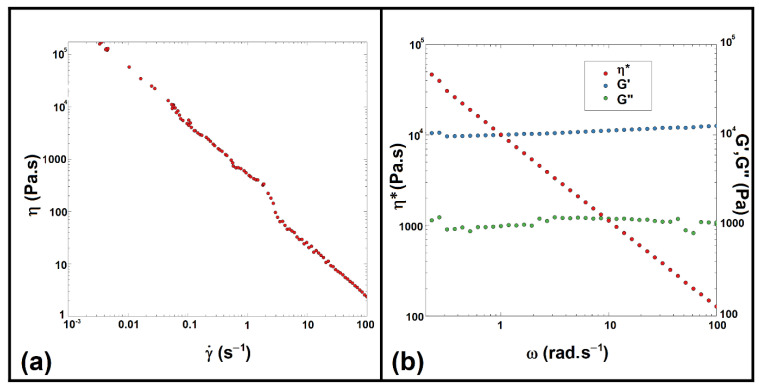
Rheological properties of xyl-TCPP-3: viscosity (h) of xyl-TCPP-3 as a function of shear rate (**a**), storage modulus (G′), and loss modulus (G″) of xyl-TCPP-3 as a function of angular frequency (**b**).

The rheological behavior of xyl-TCPP-3 is shown in Figure 7. Hydrogel viscosity was measured at 25 °C with a shear rate from 10^−3^ to 100 s^−1^. Figure 7a shows that the viscosity of xyl-TCPP-3 decreases with an increasing shear rate from above 10^5^ Pa.s to 1 Pa.s, indicating that the hydrogel exhibits shear-thinning behavior. In addition, for the oscillatory, a shear test was performed in the linear viscoelastic domain. Figure 7b shows that the value of the storage modulus (G′) is stable and larger than that of the loss modulus (G″) over the entire angular frequency range studied. Thus, this hydrogel has properties dominated by elasticity through a well-developed network, confirming its gel-like behavior [24,25].

### 2.4. In Vitro Bacterial Photoinactivation

The antibacterial activity of xyl-TCPP-3, TCPP-free, and xylan-hydrogel alone were assessed against two Gram-positive bacteria, *S. aureus* and *B. Cereus,* in the absence of irradiation and under light activation [21,22,23]. TCPP-free and xylan-hydrogel alone are used as the control. Briefly, 1 mg of Xyl-TCPP-3 was deposited in the wells of a 96-well plate and the control wells received 1 mg of xylan hydrogel alone or 50 µL of a 6.2% TCPP solution, which corresponds to the amount of TCPP brought by 1 mg of Xyl-TCPP-3. Each one of these wells received a 0.1 mL aliquot of bacterial culture in the exponential growth phase (10^6^ CFU·mL^−1^). After 30 min of incubation at 37 °C out of the light, the bacterial cultures were illuminated with white LED light for 5 h at the same temperature (total fluence of 25/cm^2^). Then, the contents of the wells were transferred to capped plastic tubes and sonicated to loosen the bacteria from the hydrogel matrices. Serial dilutions of the cell suspensions were plated on solid nutrient agar. Plate colony counts were performed after 18 h incubation at 37 °C. The results obtained are presented below (Table 2).

Xyl-TCPP-3 (as TCPP alone) showed photobactericidal activity, after illumination, against the two strains of Gram-positive bacteria (*B. Cereus* and *S. aureus)*. These results were in agreement with the production of singlet oxygen by TCCP and Xyl-TCPP-3 after illumination (Table S1). Moreover, it can be noticed that TCPP, grafted on the hydrogel, presents a reduced toxicity in the dark. Thus, the grafting of TCPP on xylan seems to decrease the toxicity of the TCCP alone. The same experiments were realized with xylan hydrogel alone (Figure S2), used as the control (CT) and obtained in a previous work [21]. Under these conditions, the two strains were not affected with or without illumination. So, the photo-antimicrobial efficiency can be attributed to the PACT effect of TCPP. These preliminary results highlight the photodynamic effect of this hydrogel against Gram-positive bacteria. Nevertheless, the concentration in PS (TCPP) is around 78 µM (higher compared to the literature [26]) and additional experiments are needed to determine the minimum amounts of TCPP that would allow, on the one hand, hydrogel formation and, on the other hand, photodynamic activity.

## 3. Conclusions

In summary, we developed PS-containing xylan-based hydrogels using TCPP as a cross-linker. The swelling tests of the obtained hydrogels showed that the xyl-TCPP-3 hydrogel functionalized with the smallest amount of TCPP possess a good swelling property. Preliminary antibacterial tests against two Gram-positive bacteria strains showed a photobacterial activity of this hydrogel only under light and the covalent grafting of TCPP on the xylan moiety seems to decrease the toxicity of the photosensitizer in the absence of light. On the other hand, the required concentration seems very important for an effective photosensitizer compared to the literature.

## 4. Materials and Methods

### 4.1. General Methods

All organic compounds, reactants, solvents, and biological nutriments were purchased from commercial compagnies and used as such. Xylan, extracted from beechwood, was purchased from Carbosynth (Compton, Berkshire, UK) and classically characterized [1]. FTIR data (600–4000 cm^−1^) were revealed by using a PerkinElmer Frontier spectrometer in ATR analysis mode. UV–Vis spectra were recorded on a SPECORD 210 double beam spectrophotometer from AnalytiK Jena company, using a 1 cm quartz cuvette. The scanning electron microscopy (SEM) analysis of the hydrogels, previously lyophilized, were realized by using a Quanta FEG 450 ESEM with an accelerating voltage of 10–15 kV. Lyophilized compounds were deposited on carbon-taped metal pins.

### 4.2. Rheological Analysis

The dynamic rheology properties were obtained by using a rotational rheometer (Mars III, ThermoFischer Scientific, RheoWin software, Waltham, MA, USA) with a 35 mm plate–plate geometry configuration with a gap of 0.500 mm. The behaviors of the gel samples were determined by the shear rate sweep tests at room temperature. Regarding the rheological properties, they were realized with strain-controlled oscillatory tests. A strain amplitude of γ_0_ = 0.05 [-] was selected to ensure a linear regime of oscillatory deformation. The hydrogel was placed between the plates at 25 °C, and the measurements were performed in the angular frequency range of 10^−1^−10^2^ rad/s.

### 4.3. Chemical Synthesis

In total, 100, 150, or 200 mg of meso-tetra(4-carboxyphenyl)porphyrin were solubilized in DMSO (13 g/L), then 8 equivalents of CDI were added. After 24 h of reaction at 60 °C, 1 g of xylan (7.57 mmol of anhydroxylose units) previously solubilized in DMSO was added. The mixture was stirred at 80 °C until a gel formed, then the stirring was stopped and the mixture was maintained at the same temperature for one day. The resulting xyl-TCPP-1, xyl-TCPP-2, and xyl-TCPP-3 hydrogels were dropped in excess distilled water. After 7 days of swelling at 25 °C, with daily water changes, the products obtained were lyophilized.

FT-IR: 3328 cm^−1^ (hydroxyl), 2918 cm^−1^, 2873 cm^−1^, 1713 cm^−1^ (carbonyl), 1603 cm^−1^ (carbonyl), 1267 cm^−1^, 800 cm^−1^, 708 cm^−1^.

### 4.4. Swelling Behavior of Hydrogels

In order to study the swelling behavior of the three xylan-TCPP hydrogels previously synthetized, a gravimetric method was used. Thus, the experiments were conducted at 37 °C with two types of aqueous solution: distilled water and PBS (pH = 7.4). The pre-weighed dry hydrogels were dropped in excess of one or the other of the two solutions. At fixed times, swollen samples were removed from the mixture with a nylon filter and they were further drained and weighed. The swelling ratio *S* at time *t* and the equilibrium swelling ratio *S_eq_* were determined as follows:

(1) $$S=\frac{W_{t}-W_{d}}{W_{d}}$$

(2) $$S_{eq}=\frac{W_{eq}-W_{d}}{W_{d}}$$

where *W_d_* is the initial weight of the dry hydrogel, and *W_t_* and *W_eq_* are the weight at time t and the equilibrium weight of the swollen hydrogel, respectively.

### 4.5. Microbial Cultures

Two Gram (+) bacterial strains *(S. aureus* CIP76.25 and *B. cereus* CH, P.a.) were cultured in liquid tryptic soy (TS) medium (pancreatic casein extract 17 g/L, soy flour papaic digest 3.0 g·L^−1^, dextrose 2.5 g·L^−1^, NaCl 5.0 g·L^−1^, and K_2_HPO_4_ 2.5 g·L^−1^) and incubated overnight aerobically at 37 °C in a rotary shaker.

### 4.6. Bacterial Photoinactivation

From a bacterial culture in log phase of growth, 100 μL of this suspension at a concentration of 10^6^ CFU/mL and 1 mg of lyophilized xyl-TCPP-3 was deposited in the wells of a 96-well plate and then incubated at 37 °C for 30 min out of the light. The plates were then illuminated with a white LED device for 5 h (with irradiance, in visible spectrum, of 1.4 mW/cm^2^). During all the illumination times, the temperature was maintained at 37 °C. Then, the well contents were transferred to eppendorfs and placed in an ultrasonic bath in order to detach all the bacteria from the hydrogel, followed by serial dilutions to 1/10. Each dilution was spread on TS + 17 g·L^−1^ agar plates using an automatic plater (easySPIRAL^®^, Interscience). After incubation at 37 °C for 24 h, colonies were counted to determine the total CFU per mL (CFU·mL^−1^). As reference, xylan hydrogel alone (Control:CT) and a solution of TCPP free at 6.2% (*w*:*v*), which corresponds to the amount of TCPP grafted on the hydrogel, was treated in the same way (referred as light control). All the experiments were performed three times in independent conditions.

## Figures and Tables

**Table 1:** Mass percentage of TCPP determined for each hydrogel.

Hydrogels	Xyl-TCPP-1	Xyl-TCPP-2	Xyl-TCPP-3
Mass percentage of TCPP after saponification of ester bond (%)	14.8	9.9	6.2

**Table 2:** Mean values of log (CFU·mL^−1^) maintained in the dark and after irradiation. CT: control; xylan-hydrogel without TCPP [21] ‘’-’’ No surviving bacteria could be detected. Nd: no reduction in cell viability was observed.

	*B. cereus*		*S. aureus*	
	Dark	Light	Dark	Light
xyl-TCPP-3	5.95	-	5.85	-
TCPP (6.2%)	4.14	-	4.1	-
CT	Nd	Nd	Nd	Nd

## Data Availability

Not applicable.

## Supplementary Materials

The following supporting information can be downloaded at: https://www.mdpi.com/article/10.3390/gels9020124/s1, Figure S1: UV–Vis standard calibration curve obtained from different concentrations of free TCPP in 2 M sodium hydroxide (λ_max_ = 410 nm); Figure S2: Photography of xylan-hydrogel free without TCPP, used as control (CT) and obtained in our previous work [21]. Table S1. Singlet oxygen quantum yield of TCPP and Xyl-TCPP-3 in DMF [23,27].
