# Supplementary material for: Xylan–Porphyrin Hydrogels as Light-Triggered Gram-Positive Antibacterial Agents
Source: Gels. 2023 Feb 2;9(2):124. doi: 10.3390/gels9020124 (PMC9957218; doi:10.3390/gels9020124)
Supplement: Supplementary file 1 [file gels-09-00124-s001.zip › gels-2149586-supplementary.pdf]

Xylan-Porphyrin Hydrogels as Light-Triggered Gram-Positive Antibacterial Agents

Abdechakour Elkihel [1], Charlotte Vernisse[1], Tan-Sothéa Ouk[1], Romain Lucas-Roper [2], Vincent Chaleix[1] and Vincent Sol [1,*]

1 Univ. Limoges, LABCiS, UR 22722, 87000 Limoges, France
2 Univ. Limoges, IRCER, UMR CNRS 7315, 87000 Limoges, France
* Correspondence: vincent.sol@unilim

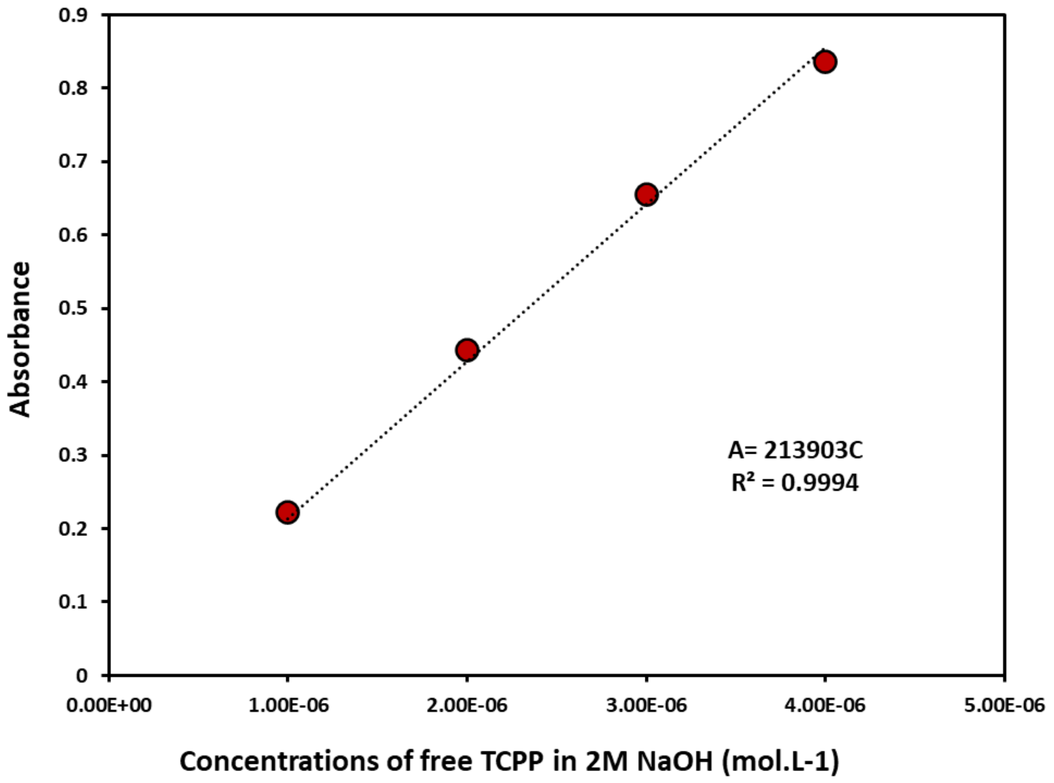

Figure S1. UV–Vis standard calibration curve obtained from different concentrations of free TCPP in 2 M sodium hydroxide (λ_{max} = 410 nm)

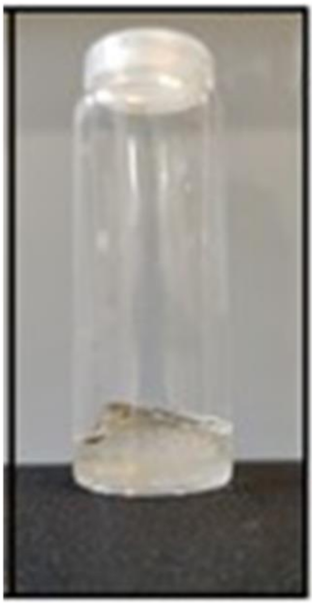

Figure S2: Photography of xylan-hydrogel free without TCPP and used as control (CT) and obtained in our previous work [21]

Table S1. Singlet oxygen quantum yield of TCPP and Xyl-TCPP-3 in DMF

Compounds	Wavelength λabs (nm)	Singlet Oxygen quantum yield Φ_Δ $^1O_2$ [a]
**TCPP**	414	0.81 ± 0.01
Xyl-TCPP	414	0.29 ± 0.01

[a] Mean of two independent experiments using tetraphenylporphyrin as standard (Φ_Δ = 0.64 in DMF) and 9,10-dimethylanthracene as decribed in supporting information [23, 27]

Determination of singlet oxygen production. Singlet oxygen generating efficiencies of porphyrins were determined by photooxidation of 9,10-dimethylanthracene (DMA) based on steady-state photolysis. H_2TPP in DMF was used as reference ($\Phi_\Delta^{Std} = 0.64$). A mixture of 1.5 mL of a stock solution of DMA in DMF (10^{-4} M) and 1.5 mL of the tested photosensitizer in DMF (10^{-6} M) was transferred into a 1 cm quartz cuvette. The solution was then saturated with air and magnetically stirred. A 150 W halogen lamp was used to irradiate the sample at a distance of 30 cm (fluence rate 0.3 mW/cm^2). A spectral window of 400-700 nm of the incident light was selected by eliminating UV and IR irradiation using an optical filter. Decrease of DMA con-centration in DMF as a function of irradiation time was monitored at 401 nm. Absence of significant spectral change ruled out any photobleaching within the experimental period. Singlet oxygen quantum yield (Φ_Δ) was calculated by using the equation below:

$$\Phi_\Delta = \Phi_\Delta^{Std} \frac{R.I_{abs}^{Std}}{R^{Std}.I_{abs}}$$

Where R and R^{Std} are the respective speeds of DMA disappearance in experimental sample and standard; $I_{abs}$and I_{abs}^{Std} are the light intensities absorbed between 400 and 700 nm by experimental sample and standard, respectively.
